# Radiation Effects on Long Period Fiber Gratings: A Review

**DOI:** 10.3390/s20092729

**Published:** 2020-05-11

**Authors:** Flavio Esposito, Anubhav Srivastava, Stefania Campopiano, Agostino Iadicicco

**Affiliations:** Department of Engineering, University of Naples “Parthenope”, Centro Direzionale Isola C4, 80143 Napoli, Italy; flavio.esposito@uniparthenope.it (F.E.); anubhav.jackie@uniparthenope.it (A.S.); campopiano@uniparthenope.it (S.C.)

**Keywords:** fiber optic sensors, long period gratings, optical fibers, radiations

## Abstract

Over the last years, fiber optic sensors have been increasingly applied for applications in environments with a high level of radiation as an alternative to electrical sensors, due to their: high immunity, high multiplexing and long-distance monitoring capability. In order to assess the feasibility of their use, investigations on optical materials and fiber optic sensors have been focusing on their response depending on radiation type, absorbed dose, dose rate, temperature and so on. In this context, this paper presents a comprehensive review of the results achieved over the last twenty years concerning the irradiation of in-fiber Long Period Gratings (LPGs). The topic is approached from the point of view of the optical engineers engaged in the design, development and testing of these devices, by focusing the attention on the fiber type, grating fabrication technique and properties, irradiation parameters and performed analysis. The aim is to provide a detailed review concerning the state of the art and to outline the future research trends.

## 1. Introduction

Beyond their typical applications for communications and sensing, optical fibers and fiber sensors have found wide interest in radiation related scenarios, due to their several advantages such as: high sensitivity and resolution measurements, low cost implementation, immunity to electromagnetic interferences, chemical inertness, long distance monitoring and high multiplexing capability [1,2,3]. Hence, there is great interest in studying ionizing and non-ionizing radiation effects on fiber optic devices to allow their use, for example, in aerospace, nuclear installations and high energy physics experiments. Based on their response, fiber optic sensors can be used for the measurement of several parameters in radiative environments if they are able to withstand the radiations, alternatively they can be used even as dosimeters by exploiting the radiation induced degradation of materials [4]. It has been observed that such effects depend on glass composition, dose rate, total dose, temperature and humidity during exposure, and post irradiation heating of the samples [5]. As a consequence, radiation induced effects on optical materials and fibers have been studied since several decades leading to a good understanding of the underlying physical mechanisms, as witnessed by several works [6,7,8,9].

Radiation can interact with materials in different forms, for example it can be distinguished between purely ionizing radiations such as gamma- and X-rays and particle radiations such as protons, neutrons, and heavy ions. The first kind delivers energy mainly through the creation of secondary electrons (and positrons), whereas the second one interacts with materials both through ionization and non-ionizing energy loss, the latter being associated for example to the displacement or the vibration of an atom [8]. Optical fiber devices have found their way for application in high energy physics experiments in data communication systems and for radiation dose and beam loss measurements in the proximity of accelerators, as for example at Compact Muon Solenoid (CMS) and ATLAS detectors in Large Hadron Collider (LHC) at European Organization for Nuclear Research (CERN). Here, ionizing radiation doses can reach hundreds of kGy and particle fluences can be up to 10^15^ cm^−2^ [10,11]. Concerning nuclear reactors, the presence of gamma radiation and neutrons is mainly envisaged. As an example, at International Thermonuclear Experimental Reactor (ITER) the neutron fluences can reach 10^18^ cm^−2^ whereas the gamma dose can accumulate up to 10 MGy [12,13], whereas in the core of a nuclear reactor even higher doses can be reached (GGy levels up to 10^20^ cm^−2^). Moreover, fiber components are also important in space applications where the main sources of energetic particles are protons and electrons in the Van Allen belts, heavy ions in the magnetosphere, cosmic ray protons and heavy ions, and protons and heavy ions from solar flares. Here, the doses are usually lower than 10 kGy [14,15]. Finally, very low doses of X-rays and protons can be found in the medicine field, ranging from tens of mGy to tens of Gy [4].

Three main physical effects can influence the working conditions of fiber-based devices when subjected to irradiation [9,16]: the radiation induced attenuation (RIA), the radiation induced refractive index (RI) change and the radiation induced emission (RIE). The fiber response is highly affected by core and cladding composition (type and concentration), due to the presence of doping elements (typically Ge, F, B, P, Al, N) introduced in the glass structure with the aim of providing light guidance and low attenuation. The RIA consists of an increase of the fiber attenuation due to radiation induced defects. Such an effect is dependent on both the irradiation parameters (dose or fluence, dose rate or flux, temperature, light power, operating wavelength) and fiber properties (geometry and composition of core and cladding, coating material, manufacturing process). The RIA usually increases when the fiber is irradiated, whereas it partially decreases when the irradiation stops and reaching a final value that is highly dependent on the temperature (e.g., the recovery is higher when temperature increases). Such kinetics is due to defect generations and bleaching mechanisms (i.e., recovery effect due to depopulation of defect center) occurring during irradiation, whereas it is due to bleaching mechanisms only in the post-irradiation phases [9]. Moreover, the refractive index changes are also very important when considering fiber sensors. In some cases, researchers found it more convenient to study the RI changes as related to two main contributions: density change through the Lorentz-Lorenz formula and induced absorption via the Kramers–Krönig relations. However, in theory, density effect is also included in the Kramers–Krönig relations, connecting the real part of permittivity (related to RI) and its imaginary part (related to absorption) [16]. Thus, in other cases RI changes are only attributed to RIA due to Kramers–Krönig [9]. Finally, the RIE effect is associated with a generation of light in the fiber core during the irradiation, which can be related to Cerenkov light in the case of high flux of sufficiently energetic particles and luminesce from precursors or radiation induced defects [9].

Until now, the most part of the studies targeted optical fibers, by mainly focusing on their radiation induced attenuation. Different fiber compositions (e.g., with Ge, B, F, Er, Al, P, Ce, Yb) were considered in order to assess the dependence of the response upon the dopant ions, and tested under different radiation conditions (transient, steady state, pulsed) and types (gamma, X-rays, neutrons, protons and so on) [16,17,18,19,20,21,22,23,24,25]. Micro-structured [26,27,28,29] and polymeric optical fibers [30,31] were also considered for the testing of radiation hardened devices and dosimeters, respectively. The fiber coating materials has also been a topic of interest [32]. Moreover, the RIE phenomenon has been employed for the development of several dosimeter configurations based on radio- and thermo-luminesce effect [33,34,35,36,37]. Afterwards, many studies and reviews also targeted Fiber Bragg Grating (FBG) sensors written in several optical fibers by different techniques and irradiated under different conditions [38,39,40,41,42,43,44,45,46,47]. Here, the attention is typically focused on the shift of the Bragg wavelength and temperature sensitivity of the gratings, as well as the influence of fiber and grating type.

Finally, despite the great interest evinced from the scientific community on the Long Period Grating (LPG) as the sensing platform [48,49,50,51,52], the number of works concerning their irradiation is lower in comparison to FBGs and currently there is no detailed review dedicated to the subject [5,43,53]. LPGs present a higher sensitivity than FBGs and they can be fabricated in several kinds of optical fibers with higher flexibility and less expensive technologies [54,55], on the other hand they are also very sensitive to bending and curvature, so additional care should be taken during measurements to avoid cross-sensitivity effects. In this context, this paper presents a thorough review of the state of the art concerning the irradiations performed on LPGs over the last twenty years. All the contributions to the topic by the research groups working in this field are reported. The attention is focused on the fiber type, grating fabrication technique and properties, irradiation parameters and performed analysis.

## 2. Irradiation of Long Period Gratings

LPG sensors are fabricated by inducing a periodic perturbation in the refractive index and/or geometry of an optical fiber. The period of the perturbation Λ typically ranges from 100–1000 µm and promotes the power coupling between the core mode and several co-propagating cladding modes [56]. As a result, the transmission spectrum of the fiber presents a series of attenuation bands located at discrete wavelengths satisfying the phase-matching condition and corresponding each one to a different cladding mode, as given by:

(1) $$\lambda_{res,i}=\left(n_{eff,co}-n_{cl,i}\right)\cdot \Lambda$$

where $n_{eff,co}$ and $n_{cl,i}$ are the effective refractive index of core and i-th cladding mode, respectively, whereas Λ is the grating period. Moreover, the depth of the attenuation bands is ruled by the product $k_{i}\cdot L$, where $k_{i}$ is the power-coupling coefficient between the core and the i-th cladding mode and *L* is the grating length. The properties of the LPG rejection bands are dependent on fiber parameters, grating properties, as well es external conditions of temperature, strain, bending and surrounding refractive index [57]. Hence, by monitoring the spectral position and/or depth of the attenuation bands, these devices can be applied in a wide range of physical, chemical and biological sensing applications [48,49,50,51,52].

Different techniques are available for their fabrication, the most important being: UV-radiation [57], CO_2_ lasers [58], IR femtosecond lasers [59], mechanical deformations [60] and electric arc discharge [61,62]. Moreover, the fiber hosting the grating affects the sensing features and can be properly selected for specialty applications, at the same time it can pose a limitation to the grating inscription techniques which can be employed.

In this section, the analysis concerning the state of the art about radiation effects on LPGs is reported taking into consideration gratings fabricated in different fibers and with different techniques. The results are primarily presented by following a chronological order and they are grouped based on the outcomes of the main research groups working in this field.

### 2.1. First Evidence of LPGs Investigated under Gamma Radiation

The first report about Long Period Gratings under gamma radiation was provided by Vasiliev et al. [63] in 1998. They irradiated an UV-induced LPG in a Ge-doped fiber (with Λ = 150 µm, coupling with HE_19_ mode) and a thermo-induced LPG in an N-doped fiber by CO-laser (Λ = 250 µm, HE_16_ mode). The gratings were exposed to a ^60^Co gamma source at a dose rate ranging from 5.4–6.6 Gy/s up to a total dose of 1.47 MGy, at 40 °C temperature. Mach–Zehnder interferometers (MZI) [64] and FBGs were also developed based on the same fibers for comparison.

The authors stated that the LPG in the N-doped fiber did not show any change in the resonance wavelength after the irradiation to within an experimental error of ±0.3 nm, the MZI response was also negligible. The same happened for the LPG in the Ge-doped fiber, differently significant phase shifts were observed in the MZI in this case. The authors justified the apparent stability of LPG response with the elimination of the precursors of gamma radiation induced color centers (i.e., crystallographic defects in material lattice that can be occupied by electrons which can absorb light) in the process of grating writing through the UV laser. Induced refractive index changes in the core region up to 2.8∙10^−5^ after a 100 kGy dose were also reported, probably due to radiation induced absorption bands of atoms in the UV region affecting the RI via the Kramers–Krönig formula. Finally, the authors stated they found some inconsistencies in their results probably due to some experimental errors.

We would like to add that the response of LPGs in the Ge-doped fiber found in [63] was unexpected, as these kinds of fibers typically present radiation-induced changes [43], as also highlighted in the following investigations reported in the next sections.

### 2.2. Systematic Study about Gamma Radiation on Chiral LPGs in Different Fibers

In [65], Henschel et al. reported the first attempt of a systematic study about the radiation effects on chiral LPGs (CLPGs), fabricated in eight different single mode fibers by Chiral Photonics, USA by twisting the fiber while passing through a miniature oven. The fibers were selected from different manufacturers and having different physical properties (resulting in low, medium and high RIA), as reported in Table 1. For those CLPGs the period Λ ranged from 589 µm to 2050 µm depending on the fiber and it was assumed to have a coupling with second, third or fourth order cladding (order of refraction N is not known precisely). The irradiations were performed by using a ^60^Co gamma source and two dose rates were considered for comparison: 0.87 Gy/s up to a total dose of 100 kGy and 0.1 Gy/s up to 20 kGy dose. Each grating was inserted into a thin quartz capillary, without fixing the fiber to have a strain-free state and mounted onto an aluminum plate.

**Figure 1:**
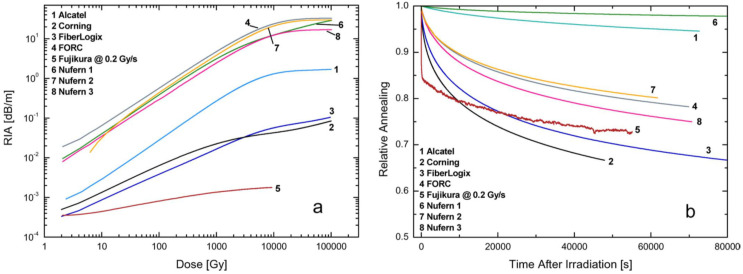
(**a**) Radiation induced attenuation in different fibers at a dose rate of 1 Gy/s and (**b**) relative recovery after the irradiation. © 2020 IEEE. Reprinted with permission from [65].

The attention was mainly focused on the fiber RIA and grating wavelength shift as a consequence of irradiation. Figure 1a,b report the fiber attenuation during the irradiation and the recovery of the same (relative, i.e., divided by the value at the end of irradiation) after the end of the irradiation, respectively. In Figure 1a it is shown that the RIA values of the radiation hardened fibers (number 2, 3 and 5) were more than 100 times lower than those of fibers 4, 6, 7 and 8.

**Figure 2:**
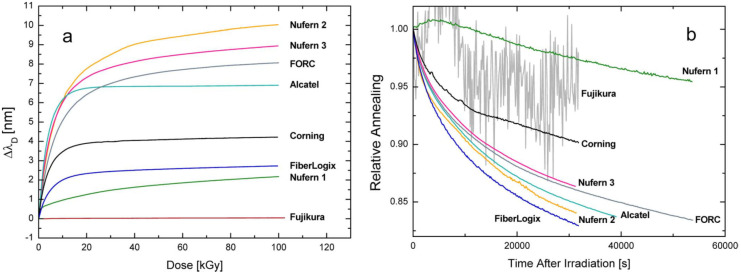
(**a**) Radiation induced wavelength shift of CLPGs at a dose rate of 0.9 Gy/s and (**b**) relative recovery after the irradiation. © 2020 IEEE. Reprinted with permission from [65].

Moreover, Figure 2a,b report the wavelength shift of the CLPGs in the same fibers as a function of irradiation dose and the recovery of the same (relative, i.e., divided by the value at the end of irradiation) after the irradiation, respectively. In Figure 2a wavelength shifts up to 10 nm can be observed after a dose of 100 kGy. The results reported in Figure 1a and Figure 2a highlight that CLPGs in the fibers with low RIA (2, 3 and 5) exhibited a lower wavelength shift, however, the shift differences were much smaller (2–3 times) than RIA differences (more than 100), except for the Fujikura fiber.

Another aspect was related to the trends of wavelength shift and RIA as a function of radiation dose. In particular, for low RIA fibers the wavelength shift saturated as the dose approached 10 kGy while the RIA still continued to increase up to 100 kGy. Differently, for high RIA fibers the behavior was the opposite: RIA saturated after 20 kGy and wavelength shift increased up to 100 kGy. Finally, for Nufern 1 (fiber 6) both RIA and wavelength shift showed a steady increase up to 100 kGy, while for Alcatel (fiber 1) they both saturated at 20 kGy. Concerning the comparison between the time dynamics of RIA and wavelength shift recovery, reported in Figure 1b and Figure 2b respectively, the situation was the following: for fiber 1 and 6 it was faster for wavelength shift than RIA, however, for the fibers 2, 3, 4 and 8 it was the opposite, whereas for fiber 7 the recovery time of RIA and resonance wavelength were similar.

Based on these results, the authors concluded that RIA and wavelength shift shows similarities but also differences; anyway it is not surprising since the RIA of most single mode fibers is primarily due to an attenuation increase of the core material, whereas the wavelength shift is dependent on radiation induced changes of both core and cladding materials (changing core and cladding effective refractive indices) through the phase-matching condition of Equation (1) and maybe also compaction (affecting grating period) has an influence.

Another interesting outcome of this research is related to the dependence of wavelength shift upon the dose rate: it was found that for a dose rate of 0.87 Gy/s it was around 1.1 to 1.2 times higher than at 0.1 Gy/s. Finally, concerning the temperature sensitivity of the CPLPGs, the authors stated that it did not change after the irradiation up to 100 kGy.

### 2.3. Gamma Radiation Sensitivity and Refractive Index Measurement Using TAP-LPG

In [66,67] Kher et al. reported about turn-around point (TAP) LPGs [57] fabricated by a CO_2_ laser in a commercial photosensitive B/Ge codoped PS980 fiber by Fibercore UK. The period of the gratings were selected in the range 206–208 µm to achieve the coupling with 11th order cladding mode and different working points in the TAP region. The LPGs were irradiated by a ^60^Co gamma source (BRIT, Gamma Chamber-900) at 1.3 kGy/h dose rate and up to a total dose of 65 kGy and measured off-line. The grating was fixed on a metal plate during irradiation.

The wavelength shifts observed in this case were the highest reported so far due to the TAP operation: each peak of the double resonance 11th mode experienced a shift (that is positive for left peak and negative for right peak) of about 35 nm after a 6 kGy dose that increased up to 80 nm when the dose reached 65 kGy. The authors attributed the wavelength shift to an increase of the core refractive index in the B/Ge codoped fiber by about 10^−5^.

The same group deepen their study in [68] by correlating the wavelength shift experimental results with numerical simulation of the phase-matching curves in order to provide a measurement of the refractive index change during the irradiation. They used the same kind of fiber (B/Ge codoped), LPG and irradiator but they reached a higher dose of 1.54 MGy. The first outcome was that the reason for wavelength shift was the radiation-induced RI change and not period (i.e., compaction), at least at high radiation doses, because those wavelength shifts would have required a tension on the fiber far beyond its breakage limit. Subsequently, under the assumption that the B/Ge fiber has an undoped silica cladding, a monotonous increase in the core refractive index with dose was found with a maximum change of 1.85·10^−4^ after a 1.54 MGy dose. This RI change recovered by less than 10^−5^ after 67 h of room temperature annealing. Finally, the authors predicted a saturation of the RI change at 8.2·10^−4^ after a 15 MGy dose.

### 2.4. Gamma Irradiation of CO_2_-Written LPGs

Kher et al. [69,70] also reported about an LPG written in an endlessly single mode (ESM) photonic crystal fiber (PCF) ESM-12-02 made by Crystal Fiber, Denmark. The grating was fabricated using a CO_2_ laser based setup, with a period of 450 µm and presented high strain sensitivity and negligible temperature response in comparison to LPGs in the standard fiber [71].

The device was tested under gamma irradiation up to 75 kGy and no significant changes were observed in spectral properties and sensing characteristics. Such fibers, along with air guiding PCFs (or photonic bandgap fibers-PBGs) have recently attracted interest in this field, as the all silica structure can lead to an improved radiation hardened response [27,28,29].

In [72,73,74] Sporea et al. performed gamma irradiation of LPGs written in an F-doped and SMF28 fiber through a CO_2_ laser assisted by a micro flame.

The F-doped model was a single mode optical fiber with 8.5 µm core diameter and fluorine concentration of 0.2 wt.% in the core and 1.8 wt.% in the cladding; the grating was fabricated by iXblue, France using a 740 µm period which resulted in the coupling with 1st order cladding mode (LP_02_) [72]. The grating region was recoated with acrylate after fabrication and was inserted into a case of glass and ceramic. The irradiation was performed by putting the LPG in the proximity of a ^60^Co industrial gamma source at “Horia Hulubei” institute (Măgurele, Romania), resulting in a dose rate of 0.2 kGy/h and up to a total dose of 45 kGy.

A blue shift of 0.7 nm was measured in the resonance wavelength after the irradiation, whereas the recovery was about 0.6 nm after 120 h at room temperature (6.7 pm/h rate). The temperature sensitivity of the grating before and after irradiation was also evaluated: it raised from 27.7 pm/°C to 29.3 pm/°C suggesting some radiation dependence of the response at high total doses.

**Figure 3:**
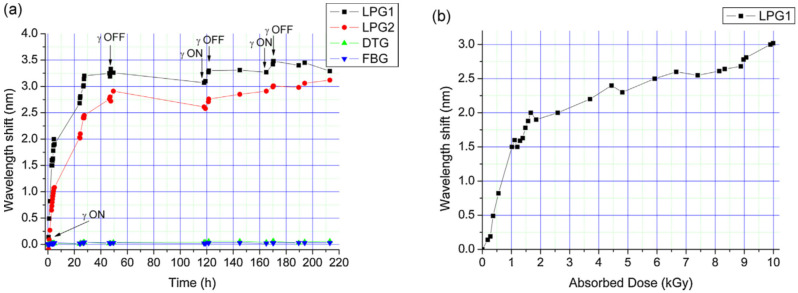
Gamma irradiation of CO_2_-written LPGs: resonance wavelength shift versus (**a**) time and (**b**) dose. © 2020 Elsevier. Reprinted with permission from [73].

A similar procedure was adopted in [73,74] for the fabrication, packaging and testing of LPGs in the SMF28 fiber. The period ranged from 720–730 µm and the 1st order cladding mode was coupled also in this case. Two LPG samples were prepared and irradiated at 0.37 kGy/h (total dose 34 kGy) and 0.24 kGy/h (total dose 21 kGy), respectively, as reported in Figure 3a.

In the first case, the final wavelength shift reached 3.3 nm after 34 kGy, however, the response saturated after the 10 kGy dose as shown in Figure 3b. In the second case, a recovery was observed at room temperature over a 211-h period (rate of 2.2 pm/h), moreover the temperature sensitivity decreased from 50 pm/°C to 48 pm/°C after a 21 kGy dose.

### 2.5. Systematic Study about Gamma and Neutron-Gamma Radiation on Arc-Induced LPGs in Different Fibers

One of the first reports about arc-induced LPGs under gamma irradiation was provided by Rego et al. [75] in 2005. They selected two pure-silica core fibers with an F-doped silica cladding, SMPS 1300-125 from Oxford Electronics (UK) and another from Acreo (SE), and they used a period of 730 µm to obtain a grating resonance around 1550 nm. The samples were irradiated using a ^60^Co gamma source at the Radio Isotope Test Arrangement (RITA) irradiation facility (SCK–CEN, Belgium) at 37.4 °C temperature, using a rate of 1 kGy/h and up to a total dose of 560 kGy. The gratings were placed into stainless-steel capillary tubes with the fiber fixed at both ends with wax. The authors did not report about wavelength shift explicitly, but they considered changes in amplitude. The experiment showed that the transmission spectra of the gratings written in both fibers remained almost unchanged. Moreover, the temperature and strain sensitivities of LPGs written in the Oxford fiber were also not affected by radiation.

Over the last years, the authors of this review have performed a systematic investigation about the effects of gamma radiation on arc-induced LPG in standard and different radiation hardened optical fibers [76,77,78,79]. In particular, the optical fibers reported in Table 2 were selected for the analysis: (i) standard Ge-doped SMF28 by Corning; (ii) Nufern R1310, which is optically similar to the standard one but with improved radiation performances [80]; (iii) Fiber-A (confidential) with doped core and silica cladding, to be used in high pressure, high temperature and corrosive environments; (iv) Fiber-B by the same manufacturer (confidential), with pure-silica core and F-doped cladding is a radiation hardened fiber. The LPGs were fabricated in the mentioned fibers by using the electric arc discharge technique due to its flexibility and possibility to be employed for several kinds of models [62,81]. The period of the gratings ranged from 625–677 µm depending on the fiber to have a resonance wavelength associated to LP_03_ or LP_04_ at 1560 nm. The irradiations were performed at room temperature at the “Horia Hulubei” institute (Măgurele, Romania) at 0.2 kGy/h dose rate, whereas the final doses ranged from 26.6–35 kGy. The gratings were kept in plastic frames to fix their strain state during irradiation and put in a thermally insulated box.

**Figure 4:**
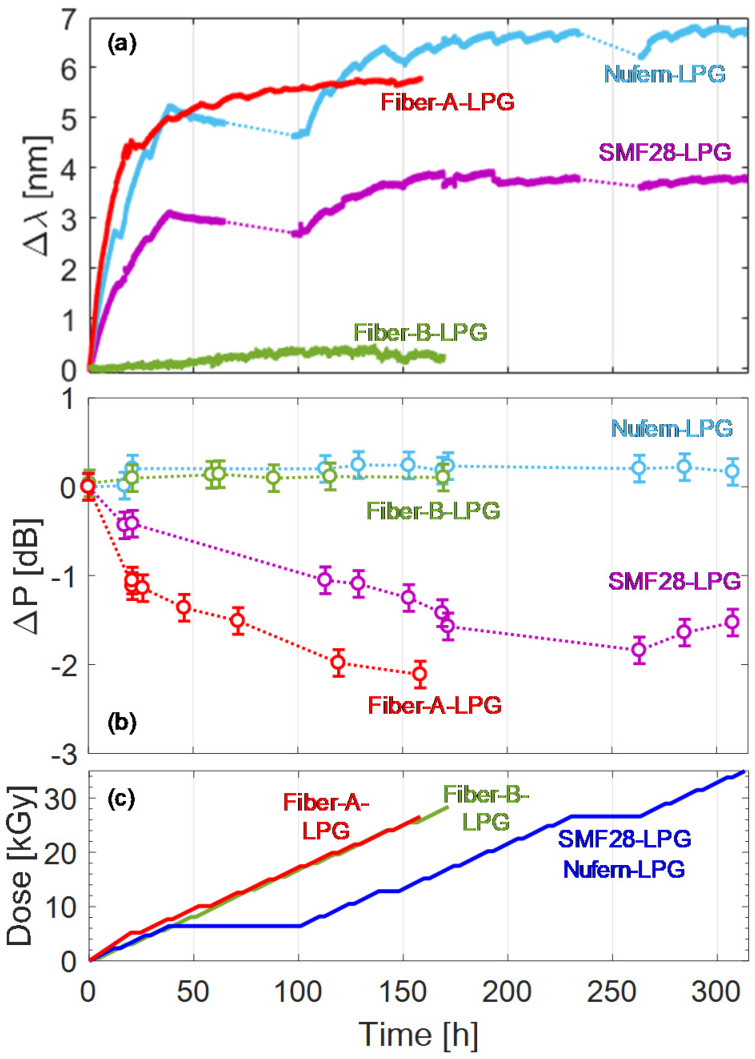
Gamma irradiation of arc-induced LPGs: (**a**) resonance wavelength shift; (**b**) transmitted power variation; (**c**) irradiation profiles. © 2020 IEEE. Reprinted with permission from [79].

The attention was focused on the real-time resonance wavelength shift and optical transmission of the fiber, as reported in Figure 4a,b, respectively. The resonance wavelengths monotonically red shifted with dose increasing (Figure 4a), showing higher rates at lower doses: for example, the LPG in Nufern fiber recorded the highest sensitivity of 1.3 nm/kGy at 0.5 kGy dose. Subsequently, a saturation of the wavelength shift was observed for doses higher than 15 kGy. At the end of the irradiation, the gratings in SMF28 and Nufern fiber exhibited wavelength shifts of 3.7 nm and 6.7 nm, respectively, after a 35 kGy absorbed dose. The grating in Fiber-A shifted of 5.7 nm as a consequence of 26.6 kGy dose, whereas the shift for Fiber-B was ~0.2 nm only despite a 29.6 kGy dose. Concerning the transmitted optical power in Figure 4b, which was measured in the range 1510–1520 nm (far from LPG bands), both SMF28 and Fiber-A showed increasing attenuation with a dose up to about 1.5 dB and 2.1 dB, respectively. Conversely for Fiber-B and Nufern the changes were quite less significant.

By combining the experimental results with full spectrum numerical simulations [82,83], the radiation induced refractive index change was also estimated: it was equal to 1.5∙10^−5^ for SMF28, 2.3∙10^−5^ for Fiber-A and Nufern in the fiber core at the end of the irradiation, whereas for Fiber-B the changes were one order of magnitude lower.

Finally, the temperature sensitivity of the gratings was also compared before and after the irradiation. For SMF28 LPG it increased from 50.5 to 53.8 pm/°C (around 6.5% change), whereas smaller variations were observed for Nufern and Fiber-A gratings, where it changed from 49.3 to 49.6 pm/°C and from 48.9 to 49.3 pm/°C, respectively. For Fiber-B LPG, the value of 22.8 pm/°C was not modified. The numerical model was also applied to estimate a change in the core thermo-optic coefficient of the SMF28 fiber of 1.5∙10^−8^ °C^−1^, whereas for the other fibers it was lower than 10^−8^ °C^−1^.

The same authors also irradiated a similar set of gratings under a mixed neutron-gamma field by using a TRIGA research nuclear reactor at the Nuclear Research Institute ICN (Mioveni, Romania) [84,85]. The fibers investigated were those reported in Table 2, i.e., SMF28, Nufern R1310, Fiber-A, in this case DrakaSRH by Prysmian-Draka was used as pure-silica core with F-doped cladding fiber. During the irradiation a gamma dose rate of 9 Gy/s was measured and a total dose of 64.8 kGy was reached after about 2 h, while the mean neutron flux was 1.25∙10^12^ n/(cm^2^∙s) resulting in a neutron fluence of 9.18∙10^15^ n/cm^2^. The gratings were kept in plastic frames to fix their strain state during irradiation and placed on an aluminum alloy plate.

**Figure 5:**
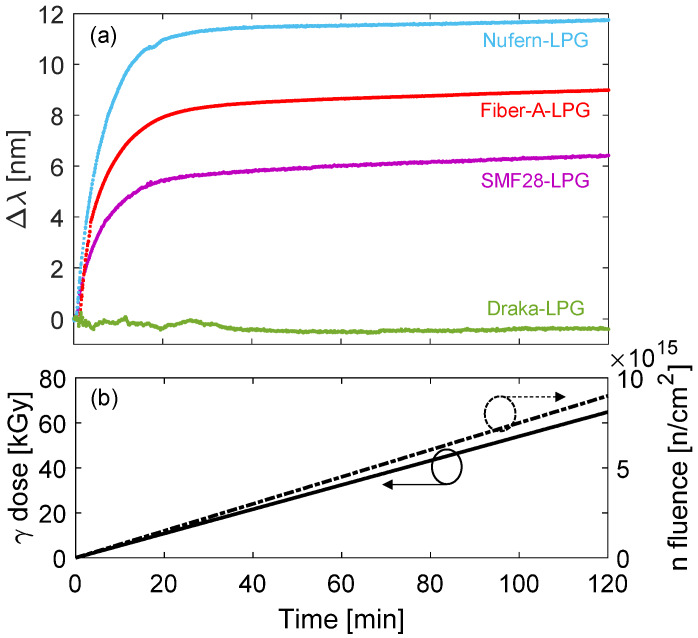
Mixed neutron-gamma irradiation of arc-induced LPGs: (**a**) resonance wavelength shift; (**b**) irradiation profiles. © 2020 Springer Nature. Reprinted from [84] (CC BY 4.0).

The real-time wavelength shift of the gratings during the irradiation is reported in Figure 5a whereas the irradiation profile is shown in Figure 5b. It was observed that the wavelength shifts monotonically increased with gamma dose and neutron fluence, the saturation was reached after about 30 min, i.e., for a gamma dose of ~16 kGy and 2.3∙10^15^ n/cm^2^ neutron fluence. The wavelength shifts recorded at the end of the irradiation were the following: 6.4 nm for LPG in SMF28, 9.0 nm for Fiber-A, 11.8 nm for Nufern and −0.4 nm for Draka. These trends were in agreement with those observed during gamma irradiation of similar samples reported in [79].

By combining the experimental results with numerical modeling, the following RI changes were estimated by the end of irradiation: 2.6∙10^−5^ for SMF28, 3.5∙10^−5^ for Fiber-A, 4.1∙10^−5^ for Nufern in the core of these fibers, whereas in the cladding of Draka the change was equal to 0.2∙10^−5^.

Finally, the temperature sensitivity of the gratings was also compared before and after the irradiation. For SMF28 and Fiber-A it increased 2% maximum, passing from the value of 50.0 and 50.8 pm/°C before the irradiation to 51.2 and 51.6 pm/°C, respectively, after the irradiation. Concerning the Nufern fiber, a greater increase in the thermal sensitivity was recorded, changing from 49.5 to 57.7 pm/°C (17% increase), whereas for Draka fiber a 10% decrease was found from 29.6 to 26.5 pm/°C. The changes in thermo-optic coefficients were also estimated by numerical analysis: in the core of SMF28 and Fiber-A they were lower than 10^−8^ °C^−1^, for the Nufern fiber it was around 3∙10^−8^ °C^−1^, finally it was 6∙10^−8^ °C^−1^ in the cladding region of Draka.

### 2.6. Proton Irradiation of LPGs

Recently, in [86,87] the group of Cusano reported the first demonstration concerning an LPG investigated under proton irradiation. For the analysis, they selected the photosensitive single mode B/Ge codoped fiber PS1250/1500 manufactured by Fibercore UK. They fabricated an LPG with a period of 308 µm using an UV laser technique. Here the coupling was with the 7th order cladding mode (LP_08_) at 1540 nm. The irradiation was performed at the CERN proton facility named IRRAD, where the grating was exposed to a proton fluence of 4.4∙10^15^ p/cm^2^ for about 6 days up to a total high dose of 1.16 MGy (dose rate of 2.36 Gy/s). The grating was mounted on a support to fix the strain conditions during the experiment.

**Figure 6:**
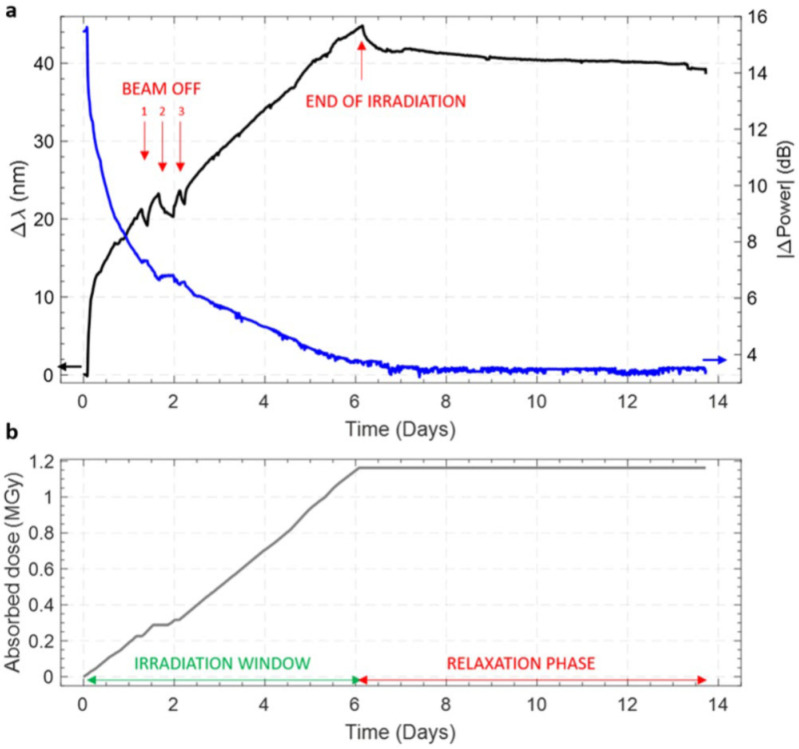
Proton irradiation of LPG: (**a**) resonance wavelength shift and peak depth change; (**b**) irradiation profile. © 2020 Springer Nature. Reprinted from [87] (CC BY 4.0).

As reported in Figure 6a, a positive wavelength shift of 44 nm was recorded in correspondence of the maximum absorbed dose, whereas the peak depth was reduced by 12 dB. The recovery phase was also observed for 7.5 days after the end of the irradiation, according to the profile in Figure 6b: a shift recovery of 6 nm was observed (corresponding to 14% of the total value) while the peak depth was kept almost unchanged from the final value at the end of irradiation.

Subsequently, the authors combined the experimental results with numerical modeling in order to estimate the changes of the main parameters affecting the grating response during the irradiation. In particular, a variation of about 1.61∙10^−4^ in the core effective refractive index was found at the end of irradiation (the recovery value was 0.15·10^−4^), while a decrease of 0.93∙10^−4^ in the grating RI modulation was estimated as well. Finally, no significant change in the temperature sensitivity of the grating was found after the irradiation.

### 2.7. Radiation Tolerant Humidity Sensors Based on LPG Coated with TiO_2_

In [88,89], the group of Cusano reported a feasibility study concerning the development of humidity sensors based on LPG technology to be applied in high energy physics experiments at CERN. For the purpose, a grating with a period of 404 µm (coupling with 5th order cladding mode) was UV-written in a B/Ge codoped fiber and it was subsequently coated with a titanium dioxide (TiO_2_) layer of about 100 nm. Such a material was selected due to its hygroscopic properties, moreover having a refractive index (n = 1.96) higher than cladding it was used to induce the mode transition phenomenon and enhance the LPG sensitivity [90,91,92].

The performance of the sensor was measured in the range 0–75% RH and at −10, 0, 10, 25 °C, to replicate the working conditions required for CERN experiments. The sensor exhibited an exponential-like response with sensitivity changing from 1.4 to 0.11 nm/%RH for humidity levels in the range 0–10% RH (at room temperature) as reported in Figure 17 from [89]. The coated LPG was thus exposed to gamma irradiation using a ^60^Co source up to a dose of 10 kGy. The preliminary results reported a wavelength shift of about 4.4 nm (at 30% RH), whereas the sensor response towards humidity exhibited the same shape (except for some variations around 16%) as a consequence of irradiation.

## 3. Discussion and Conclusions

In this work, we conducted a thorough review of the state of the art concerning the irradiations performed on LPGs over the last twenty years. We considered all the contributions to the topic by the research groups working in this field.

The first report about gamma irradiation of LPGs can be dated back to 1998 [63]. Since that work, the hosting fibers employed, the types of gratings and experimental setups have greatly increased. One of the first attempts to provide a systematic analysis was the one related to chiral LPGs fabricated in different kinds of fibers (having from low to high RIA) [65]. The attention was focused on the LPG wavelength shift and fiber RIA measurements. The results highlighted a dependence of the response upon the fiber composition and gamma dose rate, moreover it was found that wavelength shift and RIA were not always in agreement. Subsequently, TAP-LPGs were exploited in [66] to demonstrate the possibility to sense the gamma radiation with high sensitivity, as a matter of fact it is still the highest value reported so far. The same group associated the observed wavelength shift to a radiation induced refractive index change in the core of B/Ge codoped fiber at high doses, moreover they introduced the possibility to retrieve the magnitude of such changes by using simple numerical simulations [68]. On the other hand, they also tested an LPG written in PCF demonstrating a radiation hardened response [69]. Over the last five years, a larger number of works can be counted on the topic. Real-time investigations under gamma radiation of CO_2_-written LPGs in standard and radiation hardened fibers have been reported in [72,73]. Then, comparative studies about arc-induced LPGs in several standard and radiation hardened optical fibers have been reported when exposed to gamma [79] and, for the first time, to mixed neutron-gamma radiation [84]. The attention was focused on the real-time wavelength shift, peak attenuation and fiber optical power changes. Moreover the experimental results were combined with a full spectrum numerical analysis to estimate the radiation induced RI changes in the fibers. The results were strongly dependent on fiber type and were consistent between the two kinds of radiations. Finally, very recently the first report about proton irradiation of LPGs at high doses has been released [87].

For a comparative analysis, it is worth noting that most of the works attributed the shift in LPG resonance wavelengths to radiation induced refractive index changes, even under different kind of radiations and grating fabrication techniques. The shift is typically towards higher wavelengths, which can be attributed to an increase in the core RI. The amount of shift is, of course, dependent on the radiation type, dose rate, final dose, as well as fiber and grating parameters. Concerning the attenuation band depth, it seems to show trivial changes in most of experiments except for a few cases. In fact, an UV-written LPG showed a significant band decreasing under proton beam [87]. It is reasonable to believe that this effect can be attributed to the grating type rather than radiation type; the radiation induces a uniform refractive index increase that reduces the grating RI modulation. A different situation was reported in [66], where the grating experienced consistent changes in amplitude but in this case they can be attributed to the strong changes occurring in coupling coefficient in TAP region as a consequence of RI variations.

Future works should focus on the investigation of LPGs fabricated in innovative optical fibers, that have never been tested before in this field, exhibiting unconventional dopants, refractive index profiles and glass structures. In this context, the advancements in grating fabrication techniques could give a further push. Despite the huge efforts done so far and the sustained irradiation costs, there is still demand for a full understanding of the influence of dose rate (over several orders of magnitude) and irradiation temperature. Moreover, as most of the literature is focused on gamma radiation (with single reports on neutrons and protons), other kinds of radiations have to be also considered (X-rays, electrons and so on). Finally, as LPG can be integrated with sensitive overlays, the mentioned studies could be extended to coated gratings as well, for the development of different sensing devices to be used in radiation environments. The need of the hour is to develop innovative LPG sensors comprised of new features which can provide further insights into radiation effects.

## Figures and Tables

**Table 1:** Optical fibers for the fabrication of chiral LPGs (CLPGs). Data by manufacturers and authors of [65].

No.	Fiber	D_core_ (µm)	Core Dopants	Cladding Dopants
1	Alcatel 6901	8.8	GeO_2_	P_2_O_5_, GeO_2_, F
2	Corning SMF28-e	8.2	GeO_2_	None
3	FiberLogix FL-HNA-01	-	GeO_2_, F	P_2_O_5_, F
4	FORC No. 141-2	4.9	Al_2_O_3_, P_2_O_5_, GeO_2_, F	P_2_O_5_, F
5	Fujikura RR-C	8.7	F	F
6	Nufern 1 (confidential)	7.5	P_2_O_5_	P_2_O_5_, others
7	Nufern 2 (confidential)	5.25	Rare earths, others	None
8	Nufern 3 (confidential)	4.5	Rare earths, others	P_2_O_5_, others

**Table 2:** Optical fibers for the fabrication of arc-induced LPGs. Data provided by manufacturers.

No.	Fiber	D_core_ (µm)	Core Dopants	Cladding Dopants
1	Corning SMF28	8.2	GeO_2_	None
2	Nufern R1310-HTA	9.0	-	-
3	Fiber-A (confidential)	-	-	None
4	Fiber-B (confidential)	-	None	F
5	Prysmian DrakaSRH	9.0	None	F
